# Reconsultation, self-reported health status and costs following treatment at a musculoskeletal Clinical Assessment and Treatment Service (CATS): a 12-month prospective cohort study

**DOI:** 10.1136/bmjopen-2016-011735

**Published:** 2016-10-12

**Authors:** Edward Roddy, Kelvin P Jordan, Raymond Oppong, Ying Chen, Sue Jowett, Peter Dawes, Samantha L Hider, Jon Packham, Kay Stevenson, Irena Zwierska, Elaine M Hay

**Affiliations:** 1 Arthritis Research UK Primary Care Centre, Research Institute for Primary Care and Health Sciences, Keele University, Keele, Staffordshire, UK; 2 Staffordshire Rheumatology Centre, Haywood Hospital, Stoke-on-Trent, UK; 3 Health Economics Unit, School of Health and Population Sciences, University of Birmingham, Birmingham, UK; 4 Physiotherapy Department, University Hospital of North Midlands, Stoke-on-Trent, UK

**Keywords:** RHEUMATOLOGY

## Abstract

**Objectives:**

To determine (1) reconsultation frequency, (2) change in self-reported health status, (3) baseline factors associated with reconsultation and change in health status and (4) associated healthcare costs and quality-adjusted life-years (QALYs), following assessment at a musculoskeletal Clinical and Assessment Treatment Service (CATS).

**Design:**

Prospective cohort study.

**Setting:**

Single musculoskeletal CATS at the primary–secondary care interface.

**Participants:**

2166 CATS attenders followed-up by postal questionnaires at 6 and 12 months and review of medical records.

**Outcome measures:**

Primary outcome was consultation in primary care with the same musculoskeletal problem within 12 months. Secondary outcome measures were consultation at the CATS with the same musculoskeletal problem within 12 months, physical function and pain (Short Form-36), anxiety and depression (Hospital Anxiety and Depression Scale), time off work, healthcare costs and QALYs.

**Results:**

Over 12 months, 507 (38%) reconsulted for the same problem in primary care and 345 (26%) at the CATS. Primary care reconsultation in the first 3 months was associated with baseline pain interference (relative risk ratio 5.33; 95% CI 3.23 to 8.80) and spinal pain (1.75; 1.09 to 2.82), and after 3–6 months with baseline assessment by a hospital specialist (2.06; 1.13 to 3.75). Small mean improvements were seen in physical function (1.88; 95% CI 1.44 to 2.32) and body pain (3.86; 3.38 to 4.34) at 6 months. Poor physical function at 6 months was associated with obesity, chronic pain and poor baseline physical function. Mean (SD) 6-month cost and QALYs per patient were £422.40 (660.11) and 0.257 (0.144), respectively.

**Conclusions:**

While most patients are appropriate for a ‘one-stop shop’ model, those with troublesome, disabling pain and spinal pain commonly reconsult and have ongoing problems. Services should be configured to identify and address such clinical complexity.

**Strengths and limitations of this study**

- The largest study to date of outcome following treatment in a musculoskeletal Clinical and Assessment Treatment Service.
- The participation rate at baseline was high and the use of routinely collected consultation data ensured high completion rates for the primary outcome.
- Response to the postal follow-up questionnaires was poor, particularly at 12 months.
- Questionnaire length permitted inclusion of only generic measures of pain and physical function rather than body region-specific measures which might have been more sensitive to improvement.
- The study population was derived from a single geographical region and did not include a comparator cohort which might limit the generalisability of our findings.

## Introduction

Musculoskeletal problems such as osteoarthritis (OA) and back pain are highly prevalent and present frequently to primary care. One-third of adults experience low back pain annually, whereas 53% of older adults have symptomatic OA.^1^ ^2^ Annually in the UK, one-fifth of people consult their general practitioner for a musculoskeletal condition and 4% of older adults consult for OA.^3^ Musculoskeletal disorders were the largest cause of disability globally in 2013.^4^

Most of these people are managed entirely in primary care, with only a minority requiring specialist referral, traditionally to hospital-based orthopaedic and rheumatology services. Recently, patients requiring referral in the UK are increasingly managed in multidisciplinary Clinical Assessment and Treatment Services (CATS) at the primary–secondary care interface.^5^ ^6^ CATS act as a one-stop shop, providing rapid access to assessment, diagnostic investigations, treatment by appropriately skilled healthcare practitioners and onward referral pathways, aiming to provide more integrated care, and prevent chronicity, disability, and a cycle of reconsultation and referral to multiple services across primary and secondary care.^5–7^ We have previously shown that chronic pain, physical disability, anxiety, depression and work disability are prevalent among patients attending a musculoskeletal CATS, suggesting that these patients often already have chronic pain and are not being identified early, emphasising the need for appropriate early referral pathways to suitably skilled clinicians.^8^ Little is known about patient outcome following treatment in this setting and if and how patients subsequently reconsult.

The objectives of this prospective study were (1) to determine the proportion of patients reconsulting in primary care and in a musculoskeletal CATS in the 12 months following baseline assessment at the CATS, (2) to assess baseline factors associated with reconsultation in primary care and at the CATS, (3) to determine change in self-reported health status at 6 and 12 months, (4) to assess baseline factors associated with change in self-reported health status and (5) to estimate the healthcare costs and quality-adjusted life-years (QALYs) over 6 months associated with CATS attendance and determine whether these costs and QALYs differed by follow-up plan at baseline.

## Methods

This was a prospective observational study set within a musculoskeletal CATS in North Staffordshire, UK. The methods and baseline cross-sectional findings have been described previously.^8^ ^9^

### Study setting

At the time of baseline data collection, Stoke-on-Trent Primary Care Trust (PCT) served a population of >270 000 people. Referrals to secondary care musculoskeletal, rheumatology and orthopaedic services are triaged by clinicians to a multidisciplinary, musculoskeletal CATS at the primary–secondary care interface following review of referral letters, so that musculoskeletal conditions requiring non-surgical interventions are managed in the community, while appropriate cases are directed to rheumatology or orthopaedic services. The CATS is the preferred provider for patients with non-surgical, non-inflammatory musculoskeletal problems. Patients are triaged to unselected general musculoskeletal clinics within the CATS, where the type of healthcare professional patient seen (physiotherapist, rheumatologist, rehabilitation medicine specialist or GP with a special interest (GPwSI)) and the clinic in which they are seen are not determined by the index referred condition. The sole exception to this is a physiotherapist-led back pain clinic. A greater proportion of patients with back pain therefore see a physiotherapist compared to other conditions.

### Data collection

All adults aged ≥18 years seen at the CATS between February 2008 and June 2009 were invited to participate. Those who consented to take part completed a health questionnaire prior to their CATS appointment. Participants were also asked to provide consent for the research team to review their medical records, which provided data to answer the primary outcome of consultation in primary care with the same musculoskeletal problem within 12 months.

### Baseline measures

The questionnaire included physical functioning and body pain scales from the Short Form-36 (SF-36) V.2 (general population mean=50; scores <50 represent worse health).^10^ Anxiety and depression were measured using the Hospital Anxiety and Depression Scale (range 0–14; scores ≥8 on either scale representing possible or probable anxiety or depression).^11^ The presence of pain that interfered with daily activities was measured using one item from the SF-36: ‘During the past 4 weeks, how much did pain interfere with your normal work (including both work outside the home and housework)?’.^10^ Respondents answering ‘moderately’, ‘quite a bit’ or ‘extremely’ were defined as having pain interference, while responding ‘quite a bit’ or ‘extremely’ represented severe pain interference.^12–14^ Cohabitation status, self-reported height/weight, musculoskeletal pain duration and work absence in the preceding 6 months because of musculoskeletal problems were also collected. The EuroQoL-3L (EQ-5D 3L) was included, in order to calculate QALYs.^15^

The clinician conducting the CATS consultation completed a brief proforma but did not see the patient's completed questionnaire. The clinician proforma recorded the location of pain addressed in the consultation (used to group participants into four mutually exclusive categories: upper limb or neck alone, spine alone, lower limb alone or multiple sites), investigations, interventions, referrals and follow-up plan. Participants were regarded as having pain in multiple sites if the clinician recorded locations from two or more of the upper limb/neck, spine or lower limb, or recorded a diagnosis of fibromyalgia, chronic widespread pain, generalised OA or polymyalgia rheumatica. Follow-up was categorised as referral to other services (eg, rheumatology, orthopaedics, physiotherapy), CATS follow-up, discharge to the GP or to be decided following investigations. If the follow-up plan was dependent on investigation results, follow-up information for those participants who consented to medical record review was extracted from CATS records. The clinician profession was also recorded. Owing to the low number of rehabilitation medicine specialists, we combined these with rheumatologists (referred to hereafter as hospital specialists). We also recorded whether patients were attending a general musculoskeletal clinic or the physiotherapist-led back pain clinic described above.

### Follow-up questionnaires

A self-administered questionnaire containing the same measures as at baseline was mailed at 6 and 12 months to all consenting participants. Non-responders were sent a postcard reminder after 2 weeks and a repeat questionnaire after 4 weeks.

### Medical record review

In participants consenting to medical record review, information was extracted from primary care records for the 12 months following baseline. Owing to the large number of general practices (n=114 including 49 with fewer than 10 patients), it was unfeasible to examine the records of all patients and so a pragmatic decision was made to extract records from the 57 most accessible practices with the highest number of participants. Records were downloaded electronically where possible but where software was incompatible with the practice, information was extracted manually using a proforma. The information extracted was date of any musculoskeletal consultation in primary care, and date of a musculoskeletal consultation for the same body location (neck, shoulder, elbow, hand/wrist, spine, hip, knee, foot/ankle) as recorded by the clinician at baseline. Musculoskeletal consultations were identified using the Read code system which is commonly used to record morbidity in UK primary care.^16^ Free-text narrative data were not extracted from the medical record. We identified the date of any further attendance at the CATS for the same body location in all participants who consented to medical record review through manual review of CATS records.

### Sample size

At the time of baseline data collection, ∼3500 patients were seen in the CATS annually. Based on previous studies, we expected 75% of these to participate at baseline (n=2625), and 75% of these to separately consent to further postal contact and medical record review (n=2000 each). While we aimed to review records of as many patients as feasible, as an example, a sample size of 1125 is sufficient to determine the percentage making a repeat primary care consultation during 12 months follow-up with a margin of error of 3% and a 95% confidence level, based on an estimate of 50%.

### Statistical analysis

We compared baseline responders with extracted medical record data with all other baseline responders on baseline sociodemographic, pain characteristics and general health. The percentage consulting for a musculoskeletal problem in primary care in the 12 months after baseline was determined. The primary analysis was based on the time to consulting in primary care about the same body location which was addressed at the baseline CATS consultation. We split time to first consultation to primary care into no consultation during 12-month follow-up, first consultation within 3 months (early), between 3 and 6 months, and between 6 and 12 months after baseline clinic assessment (late). We used these categories rather than actual time as it was evident that the baseline factors associated with first primary care attendance changed over the 12-month time-period, and it was considered that attendance to primary care within 3 months may be at the request of the CATS clinician. Multinomial logistic regression was used to determine the association of follow-up plan and clinician profession with the time of consultation to primary care adjusted for self-reported (age, gender, cohabitation, pain interference and duration, body mass index, anxiety and depression) and clinician-reported (body region, musculoskeletal or back pain clinic) factors. No repeat consultation was treated as the reference category. Results are reported as adjusted relative risk ratios (RRR) with 95% CIs.

Secondary outcomes were reconsultation at the CATS during 12-month follow-up and self-reported health (physical function, body pain, anxiety and depression, and time off work in those employed at baseline) at 6 and 12 months. Binary logistic regression was used to assess association of clinician profession and follow-up plan with reconsultation at the CATS about the same body location as at the baseline clinic at any point during the 12 months, adjusting for the same factors included in the primary outcome analysis. Results are presented as ORs with 95% CI. Multiple linear regression was used to assess the association of clinician profession and follow-up plan with physical function score at follow-up adjusted for baseline score and for the same baseline self-reported and clinician-reported factors as included in the analysis of primary care consultation (except pain interference as it was highly correlated with baseline physical function).

Two sensitivity analyses were performed. First, as primary care medical record information was not available for everyone, we performed multiple imputation with 50 imputations and again repeated the analysis. Second, because of the attrition at follow-up, the analysis of self-reported physical function at 6 months was repeated using multiple imputed data for those not responding at follow-up.

### Analysis of healthcare costs and QALYs

The health economic analysis was conducted from a healthcare perspective and focused on estimating the costs and QALYs arising from attending the CATS. Resource use data were collected from the clinician proforma and 6-month questionnaire. The proforma recorded investigations and interventions that patients received, while the questionnaire asked about the number and type of health professionals seen, medication taken and the number of interventions. Unit costs for individual resource use items were obtained from sources such as the British National Formulary (BNF), Personal Social Services Research Unit and NHS reference costs.^17–19^ The analysis was limited to those who completed the questionnaire. In order to value the resource use items, we multiplied resource use by unit costs and estimated a total cost per patient by summing up the costs associated with each resource use item. The area under the curve approach was used to estimate QALYs using EQ-5D responses at baseline and 6 months. Multiple regression was used to estimate mean total cost and QALYs by follow-up plan controlling for body region, age, body mass index (BMI), anxiety and depression, pain interference and baseline EQ-5D. Bootstrapping (1000 replications) was used to estimate bias-corrected CIs around differences in mean costs and QALYs between groups using patients who were referred to other specialities as the reference category.

## Results

As reported previously,^8^ 3429 patients were mailed the baseline questionnaire of whom 453 (13%) did not attend their CATS appointment. Two thousand one hundred and sixty-six consented to participate at baseline, from whom 2116 clinician proformas were completed (adjusted response 71%). Of these, 1453 (69%) had their medical records reviewed and did not have a primary care musculoskeletal consultation on the same day as their CATS appointment (see online supplementary figure S1). Compared to those responding but not undergoing record review, these participants were older (mean difference 2.6 years, 95% CI 1.3 to 4.0) and had slightly worse levels of pain, but no differences on gender, anxiety, depression, physical functioning, pain duration, pain interference, employment status or time off work (see online supplementary table S1).

10.1136/bmjopen-2016-011735.supp1 supplementary figure Flow of participants through the study

10.1136/bmjopen-2016-011735.supp2 supplementary table comparison of baseline characteristics between those included and excluded from primary care record analysis

### Consultation in primary care during 12-month follow-up

Of the 1453 for whom record data were collected, 1342 were included in the primary outcome analysis as the remainder received other diagnoses such as gout, inflammatory arthritis and joint hypermobility, and hence, a specific body region was not available to link subsequent consultations to (see online supplementary figure S1). Of these, 507 (38%, 95% CI 35% to 40%) consulted primary care during 12-month follow-up for the same body region as addressed at the baseline clinic assessment. Median number of days to consulting primary care was 69 (IQR 27 to 159): 289 (22%) consulted within 3 months and 403 (30%) within 6 months. There was no association between the type of professional seen at baseline and consulting in the first 3 months but those seeing a hospital specialist were more likely to first return to primary care between 3 and 6 months after their CATS visit (adjusted RRR 2.06; 95% CI 1.13 to 3.75 compared to GPwSI) and between 6 and 12 months (2.08; 1.12 to 3.88) (table 1). The strongest association with consulting within the first 6 months was with severe pain interference at baseline (within 3 months: 5.33; 3.23 to 8.80 compared to no pain interference; 3–6 months: 2.26; 1.25 to 4.09). Those consulting with a spine problem were more likely to consult primary care in the first 3 months (1.75; 1.09 to 2.82) or after 6–12 months (2.17; 1.06 to 4.47) compared to having an upper limb or neck problem. Those with anxiety or depression were less likely to first consult primary care between 6 and 12 months (0.60; 0.38 to 0.95). There was no association of gender, cohabitation status, follow-up plan, pain duration or BMI with primary care consultation.

**Table 1:** Associations with time after baseline assessment of first primary care consultation for musculoskeletal problem in same body location as at baseline CATS consultation

		0–3 months (early)	3–6 months	6–12 months (late)
Total consulting (% consulting)	507 (38)	289 (22)	114 (8)	104 (8)
	n (% consulting)	Adjusted RRR* (95% CI)		
Male	586 (37)	1.00	1.00	1.00
Female	756 (38)	1.05 (0.79 to 1.42)	0.96 (0.64 to 1.46)	0.78 (0.51 to 1.20)
Age (years)				
18–44	424 (37)	1.00	1.00	1.00
45–64	633 (38)	1.11 (0.80 to 1.55)	1.11 (0.68 to 1.81)	1.03 (0.63 to 1.70)
≥65	285 (38)	0.92 (0.60 to 1.41)	1.33 (0.75 to 2.36)	1.38 (0.77 to 2.48)
Living alone				
No	1140 (38)	1.00	1.00	1.00
Yes	202 (39)	0.76 (0.49 to 1.18)	1.33 (0.79 to 2.23)	1.40 (0.80 to 2.45)
Professional seen				
GPwSI	309 (29)	1.00	1.00	1.00
Hospital specialist	359 (39)	1.25 (0.82 to 1.92)	**2.06 (1.13 to 3.75)**†	**2.08 (1.12 to 3.88)**†
Physiotherapist	674 (41)	1.16 (0.77 to 1.73)	1.58 (0.88 to 2.84)	1.40 (0.76 to 2.60)
Region at clinic				
Upper limb/neck	436 (32)	1.00	1.00	1.00
Spine	347 (52)	1.75 (1.09 to 2.82)†	1.12 (0.56 to 2.24)	**2.17 (1.06 to 4.47)**†
Lower limb	454 (31)	0.87 (0.60 to 1.28)	0.86 (0.53 to 1.41)	1.38 (0.81 to 2.36)
Multiple regions	105 (42)	1.16 (0.66 to 2.04)	0.97 (0.44 to 2.14)	1.77 (0.78 to 4.02)
Follow-up plan				
Referred	492 (38)	1.00	1.00	1.00
Followed-up	145 (47)	1.28 (0.80 to 2.06)	1.28 (0.65 to 2.50)	0.86 (0.36 to 2.10)
Discharged	637 (35)	0.95 (0.69 to 1.30)	0.75 (0.48 to 1.17)	1.39 (0.87 to 2.21)
Unknown	68 (46)	1.40 (0.71 to 2.75)	1.29 (0.53 to 3.13)	1.87 (0.79 to 4.46)
Pain duration				
<12 months	603 (37)	1.00	1.00	1.00
>12 months	738 (38)	0.80 (0.60 to 1.07)	0.91 (0.60 to 1.37)	1.28 (0.82 to 1.98)
Pain interference				
No/little bit	300 (22)	1.00	1.00	1.00
Moderately	295 (29)	**2.38 (1.37 to 4.14)**†	1.07 (0.54 to 2.13)	0.72 (0.38 to 1.37)
Quite a bit/extremely	746 (47)	**5.33 (3.23 to 8.80)**†	**2.26 (1.25 to 4.09)**†	1.35 (0.78 to 2.33)
BMI				
Normal	382 (35)	1.00	1.00	1.00
Overweight	484 (36)	1.18 (0.82 to 1.71)	0.77 (0.46 to 1.30)	1.07 (0.61 to 1.86)
Obese	439 (42)	1.28 (0.88 to 1.87)	1.16 (0.70 to 1.93)	1.63 (0.95 to 2.82)
Unknown	37 (38)	0.89 (0.35 to 2.23)	1.37 (0.47 to 3.94)	0.86 (0.19 to 3.97)
Anxious/depressed				
No	606 (34)	1.00	1.00	1.00
Yes	736 (41)	1.02 (0.75 to 1.40)	0.97 (0.62 to 1.50)	0.60 (0.38 to 0.95)†

Bold typeface denotes statistical significance.

Percentages are row percentages.

*Multinomial logistic regression n=1340 (two participants omitted due to missing data). RRR adjusted for all presented variables and type of clinic attended (general musculoskeletal or physiotherapist-led back pain clinic), reference group is no musculoskeletal consultation.

†p<0.05.

BMI, body mass index; CATS, Clinical and Assessment Treatment Service; GPwSI, general practitioner with a special interest; RRR, relative risk ratios.

Analysis based on multiple imputation data yielded similar estimates and CIs to those from the analysis for those whose records were reviewed.

### Reconsultation at the CATS during 12-month follow-up

Three hundred and forty-five (26%) reconsulted at the CATS during 12-month follow-up for a musculoskeletal problem in the same body location as assessed at baseline. The clinician stating they would follow-up the patient (adjusted OR 9.97; 95% CI 6.36 to 15.62) was most strongly associated with reconsultation at the CATS while being discharged (1.54; 1.13 to 2.10) was also significantly associated (table 2). Patients seeing a hospital specialist were more likely to reconsult in the CATS (OR 1.68; 95% CI 1.14 to 2.49 compared to GPwSI). Severe pain interference and shorter pain duration were also associated with reconsultation.

**Table 2:** Associations with return to interface clinic during 12-month follow-up for musculoskeletal problem in same body location as baseline CATS consultation

	Total n (% with appointment)	OR (95% CI)	Adjusted OR* (95% CI)
Total	1342 (26)		
Male	586 (25)	1.00	1.00
Female	756 (26)	1.07 (0.84 to 1.28)	1.10 (0.83 to 1.45)
Age (years)			
18–44	424 (23)	1.00	1.00
45–64	633 (27)	1.23 (0.93 to 1.64)	1.35 (0.98 to 1.86)
≥65	285 (27)	1.21 (0.86 to 1.71)	1.41 (0.95 to 2.09)
Living alone			
No	1140 (26)	1.00	1.00
Yes	202 (23)	0.83 (0.58 to 1.18)	0.89 (0.60 to 1.32)
Professional seen			
GPwSI	309 (23)	1.00	1.00
Hospital specialist	359 (26)	1.15 (0.81 to 1.64)	**1.68 (1.14 to 2.49)**†
Physiotherapist	674 (27)	1.20 (0.88 to 1.64)	0.91 (0.62 to 1.34)
Region at clinic			
Upper limb/neck	436 (27)	1.00	1.00
Spine	347 (35)	**1.48 (1.09 to 2.01)**†	0.84 (0.53 to 1.35)
Lower limb	454 (17)	**0.54 (0.39 to 0.75)**†	**0.59 (0.41 to 0.83)**†
Multiple regions	105 (30)	1.14 (0.71 to 1.83)	1.05 (0.63 to 1.77)
Follow-up plan			
Referred	492 (17)	1.00	1.00
Followed-up	145 (68)	**10.61 (6.96 to 16.17)**†	**9.97 (6.36 to 15.62)**†
Discharged	637 (23)	**1.50 (1.12 to 2.03)**†	**1.54 (1.13 to 2.10)**†
Unknown	68 (21)	1.28 (0.68 to 2.41)	1.19 (0.62 to 2.29)
Pain duration			
<12 months	603 (27)	1.00	1.00
>12 months	738 (24)	0.85 (0.66 to 1.09)	**0.75 (0.57 to 0.99)**†
Pain interference			
No/little bit	300 (19)	1.00	1.00
Moderately	295 (25)	**1.49 (1.00 to 2.20)**†	1.53 (1.00 to 2.35)
Quite a bit/extremely	746 (29)	**1.75 (1.26 to 2.44)**†	**1.64 (1.11 to 2.42)**†
BMI			
Normal	382 (25)	1.00	1.00
Overweight	484 (26)	1.03 (0.76 to 1.41)	1.10 (0.78 to 1.55)
Obese	439 (26)	1.06 (0.77 to 1.44)	1.12 (0.79 to 1.58)
Unknown	37 (16)	0.57 (0.23 to 1.40)	0.45 (0.17 to 1.22)
Anxious/depressed			
No	606 (25)	1.00	1.00
Yes	736 (26)	1.06 (0.83 to 1.36)	0.92 (0.68 to 1.23)

Bold typeface denotes statistical significance.

Percentages are row percentages.

*Binary logistic regression n=1340 (two participants omitted due to missing data). Adjusted for all presented variables and type of clinic attended (general musculoskeletal or physiotherapist-led back pain clinic), referent group is no follow-up appointment.

†p<0.05.

BMI, body mass index; CATS, Clinical and Assessment Treatment Service; GPwSI, general practitioner with a special interest.

### Self-reported health and time off work

One thousand one hundred and forty-three (54%) of the 2116 baseline responders with a completed clinician proforma completed the follow-up questionnaire at 6 months and 762 (36%) at 12 months. Six-month responders were older (mean age 54.9 vs 46.4) compared to non-responders and had lower levels of anxiety and depression. However, they did not differ according to pain, physical function or the type of clinician seen at baseline. Responders showed some improvement at 6 months in body pain (mean change 3.86; 95% CI 3.38 to 4.34; effect size equivalent 0.47), whereas a smaller change was seen in physical functioning (mean change 1.88; 95% CI 1.44 to 2.32; effect size equivalent 0.16) (table 3). The percentage with severe pain interference fell from 54% to 40% at 6 months, while the percentage taking time off work due to their musculoskeletal problem fell from 42% to 33%. However, there was no change in anxiety or depression levels, nor was there any further change in any of these measures at 12 months. Given the high attrition at 12 months, and the lack of change at the population level between 6 and 12 months, the remainder of the self-reported analysis concentrated on the 6-month time-point.

**Table 3:** Self-reported change in physical and mental health status, time off work and pain interference at 6 and 12 months

	Baseline*	6 months	12 months
	Mean (SD)	Mean change† (95% CI)	Mean change† (95% CI)
HADS depression	6.1 (4.2)	−0.02 (−0.20 to 0.16)	−0.13 (−0.35 to 0.09)
HADS anxiety	7.5 (4.6)	0.03 (−0.16 to 0.22)	−0.02 (−0.26 to 0.22)
SF-36 physical function	36.4 (11.9)	**1.88 (1.44 to 2.32)**	**1.82 (1.24 to 2.39)**
SF-36 body pain	34.5 (8.3)	**3.86 (3.38 to 4.34)**	**4.19 (3.55 to 4.83)**
	n (%)	n (%)	n (%)
Time off work‡	219 (42)	169 (33)	110 (33)
Anxious/depressed	588 (53)	591 (53)	390 (52)
Pain interference			
Not at all/a little	258 (23)	429 (38)	284 (38)
Moderately	261 (23)	247 (22)	178 (24)
Quite a bit/extremely	606 (54)	449 (40)	289 (38)

Bold typeface denotes statistical significance.

Number in analysis for baseline and 6 months: 1118 (depression), 1115 (anxiety), 1124 (physical function), 1109 (body pain), 519 (time off work), 1115 (anxiety/depression), 1125 (interfering pain). Number in analysis for 12 months: 748 (depression), 748 (anxiety), 754 (physical function), 745 (body pain), 332 (time off work), 748 (anxiety/depression), 751 (interfering pain).

*In those responding at 6 months.

†Positive mean change indicates improvement.

‡In those currently employed at baseline n=519. At 6 and 12 months, time off work includes those no longer employed.

HADS, Hospital Anxiety and Depression Scale; SF-36, Short Form-36.

Type of clinician seen and follow-up plan did not associate with physical function at 6 months (table 4). Females, older adults, those who were obese, those with pain duration of >12 months and those with worse physical function at baseline had the worst outcomes at 6 months. For example, those with pain duration longer than 12 months at baseline had a mean physical functioning score at 6 months around 2 points worse than those with shorter pain duration (adjusted mean difference −1.93; 95% CI −2.83 to −1.03). Sensitivity analysis based on multiple imputation data made little difference to these estimates and CIs.

**Table 4:** Six-month follow-up PF score by baseline factors

	Adjusted for baseline PF score only Coefficient (95% CI)	Adjusted for all variables Coefficient (95% CI)
Female (referent: male)	−0.84 (−1.73 to 0.06)	−0.90 (−1.80 to 0.00)*
Age (years) (referent: 18–44)		
45–64	−1.41 (2.50 to −0.33)	−1.27 (−2.36 to −0.17)*
≥65	−2.94 (−4.21 to −1.67)*	−2.87 (−4.19 to −1.55)*
Living alone (referent: not living alone)	−1.14 (−2.37 to 0.09)	−0.53 (−1.77 to 0.72)
Professional seen (referent: GPwSI)		
Hospital specialist	0.05 (−1.17 to 1.27)	−0.24 (−1.45 to 0.97)
Physiotherapist	0.69 (−0.40 to 1.77)	−0.32 (−1.48 to 0.84)
Region at clinic (referent: upper limb/neck)		
Spine	2.02 (0.82 to 3.21)*	0.52 (−1.02 to 2.05)
Lower limb	−0.05 (−1.20 to 1.10)	−0.47 (−1.62 to 0.68)
Multiple regions	−0.34 (−1.89 to 1.20)	−0.58 (−2.13 to 0.96)
Other	0.07 (−2.10 to 2.23)	−0.72 (−2.87 to 1.43)
Follow-up plan (referent: referred)		
Followed-up	0.05 (−1.45 to 1.56)	−0.91 (−2.45 to 0.63)
Discharged	0.23 (−0.75 to 1.21)	0.12 (−0.85 to 1.09)
Unknown	−0.48 (−2.26 to 1.30)	−0.58 (−2.33 to 1.17)
Pain duration >12 months (referent <12 months)	−1.99 (−2.90 to −1.09)*	−1.93 (−2.83 to −1.03)*
BMI (referent: normal BMI)		
Overweight	0.30 (−0.77 to 1.36)	0.17 (−0.89 to 1.22)
Obese	−1.59 (−2.75 to −0.43)*	−1.58 (−2.74 to −0.43)*
Unknown	−1.65 (−4.38 to 1.08)	−1.09 (−3.80 to 1.63)
Anxious/depressed (referent: not anxious/depressed)	−0.65 (−1.59 to 0.29)	−0.81 (−1.75 to 0.12)
Baseline PF score†	0.87 (0.82 to 0.92)*	0.79 (0.75 to 0.84)*

Bold typeface denotes statistical significance.

Coefficient is adjusted mean difference in PF score at follow-up compared to referent, with positive coefficient indicating higher (better) PF score, complete data only, n=1124.

*p<0.05.

†Per unit PF score at baseline.

BMI, body mass index; GPwSI, general practitioner with a special interest, PF, physical functioning.

### Healthcare costs and QALYs

The overall mean (SD) cost per patient incurred by attending the CATS was £422.40 (660.11) over the 6-month period. The mean cost associated with patients who were discharged was significantly lower than those who were referred to other specialities (mean difference £−132.57; 95% CI −226.78 to −49.54) (table 5). Costs associated with patients in the other groups (followed-up and unknown) were not significantly different from costs associated with patients who were referred (table 5).

**Table 5:** Mean costs and QALYs over 6 months according to follow-up plan

	Mean cost*	Mean difference (95% CI)†	Mean QALYs*	Mean difference (95% CI)†
Referred (n=405)	£497.55		0.2572	
Followed-up (n=124)	£437.18	£−60.37 (−172.68 to 67.36)‡	0.2566	−0.001 (−0.014 to 0.014)‡
Discharged (n=526)	£364.98	£−132.57§ (−226.78 to −49.54)‡	0.2591	0.002 (−0.006 to 0.009)‡
Unknown (n=82)	£397.23	£−100.32 (−227.19 to 93.92)‡	0.2498	−0.007 (−0.020 to 0.005)‡

*Values are predicted means obtained from multiple regression controlling for body region, age, body mass index, anxiety, depression, pain interference and baseline EQ-5D.

†Compared to patients who were referred.

‡Bootstrapped CI.

§p<0.05.

QALYs, quality-adjusted life-years.

Across all participants, the mean (SD) QALYs per patient over the 6-month period was 0.257 (0.144). There was no significant difference in mean QALYs between patients who were referred to other specialities and any other group.

## Discussion

After assessment in a musculoskeletal CATS, nearly 40% of people consulted primary care about the same problem within 12 months, with over half of these consulting within 3 months. Similarly, a quarter of patients reconsult in the CATS within 12 months. People with pain interference and spinal pain were more likely to reconsult. Over 6 months, only small changes were seen in body pain and physical function, and in the proportion reporting pain interference and taking time off work because of their musculoskeletal problem. Functional outcome was worst in those with older age, obesity, chronicity and pre-existing physical impairment. The cost-outcome description found that follow-up plan to see again in the CATS or to refer to another specialty attracted higher mean costs.

The explanations for frequent reconsultation are likely to be multifactorial, and our study design cannot elucidate these. It may be that patients were advised to visit their GP for change in medication, they reconsulted to obtain a repeat prescription, the CATS consultation failed to adequately meet patients’ expectations and/or their symptoms did not improve. The observation that patients assessed by hospital specialists were more likely to reconsult could be explained by specialists advising further consultation in primary care, for example, to change medication, rather than this reflecting poor outcome. The findings that people with pain interference (either setting) and spinal pain (primary care) were more likely to consult and worse functional outcome was associated with older age, obesity, chronicity and pre-existing physical impairment suggest that the current model of care does not meet the needs of those with the most troublesome symptoms. An unexpected finding that we find difficult to explain was that people with anxiety or depression were less likely to first consult primary care between 6 and 12 months.

There are few suitable cohorts to compare our findings to. In a study undertaken in a physiotherapist-led CATS, small improvements were reported in pain and general health (EQ-5D) over 12 months but no change was seen in the SF-36.^20^ Most improvement occurred within 3 months but was less likely in people with spinal pain and chronic symptoms. Repeat consultation was not examined. Several studies have found similar rates of repeat musculoskeletal consultations in primary care following an initial primary care consultation. One-third to one-half of primary care shoulder pain consulters in Scandinavia reconsult within 12 months.^21^ ^22^ We have previously shown that 34% of knee pain consulters and 22% of foot pain consulters consult again in primary care with the same problem over 18 months.^23^ ^24^

This is the largest study to date of outcome following treatment in musculoskeletal CATS. Strengths of the study are the high participation rate at baseline (73%) and the use of routinely collected consultation data to ensure high completion rates for the primary outcome. Several limitations are, however, worthy of further discussion. First, the response to the postal follow-up questionnaires was poor, particularly at 12 months. However, questionnaire data were used to answer the secondary objectives rather than the primary objective which used data from consultation records, available for 69% of baseline responders. Furthermore, a sensitivity analysis using multiple imputation to account for loss to follow-up did not significantly alter our findings. Second, pain and physical function were measured using generic health status instruments, finding only small changes over time. Owing to questionnaire length, we could not include body region-specific questionnaires which might have been more sensitive to improvement. Third, the study population was derived from a single geographical region and participants recruited from a single CATS which might limit the generalisability of our findings. Finally, we did not include a comparator cohort to allow a direct comparison to patients managed in other settings such as primary care, orthopaedics or rheumatology.

Our findings suggest that musculoskeletal CATS should be configured to address troublesome disabling pain since it is patients with the most bothersome symptoms who are most likely to reconsult either in primary care or at the CATS and to experience poor functional outcome. We have previously highlighted the complexity of patients referred from primary care to musculoskeletal CATS, showing chronic pain, major physical limitation, anxiety, depression and work disability to be highly prevalent.^8^ Our finding that poor outcome is associated with pain interference, obesity, pain duration and physical impairment raises the possibility that targeting specific treatments at people with certain modifiable risk factors might improve outcome, as has been shown to be the case in other settings, for example, stratified care using the STarT Back tool in people with low back pain in primary care.^25^ However, further research is needed to determine how to identify people at risk of poor outcome from musculoskeletal problems and evaluate what targeted treatment should consist of. Notwithstanding this important future research agenda, we suggest that musculoskeletal services need to be resourced to provide a biopsychosocial model of care, with appropriately trained clinical staff, and that services need the flexibility and resource to offer follow-up appointments, where clinically indicated, in order to monitor progress, tailor treatment to the individual and address clinical complexity.
