# Supplementary figures and images for: Reconsultation, self-reported health status and costs following treatment at a musculoskeletal Clinical Assessment and Treatment Service (CATS): a 12-month prospective cohort study
Source: BMJ Open. 2016 Oct 12;6(10):e011735. doi: 10.1136/bmjopen-2016-011735 (PMC5073523; doi:10.1136/bmjopen-2016-011735)

## Supplementary Figure

Supplementary Figure – Flow of participants through the study

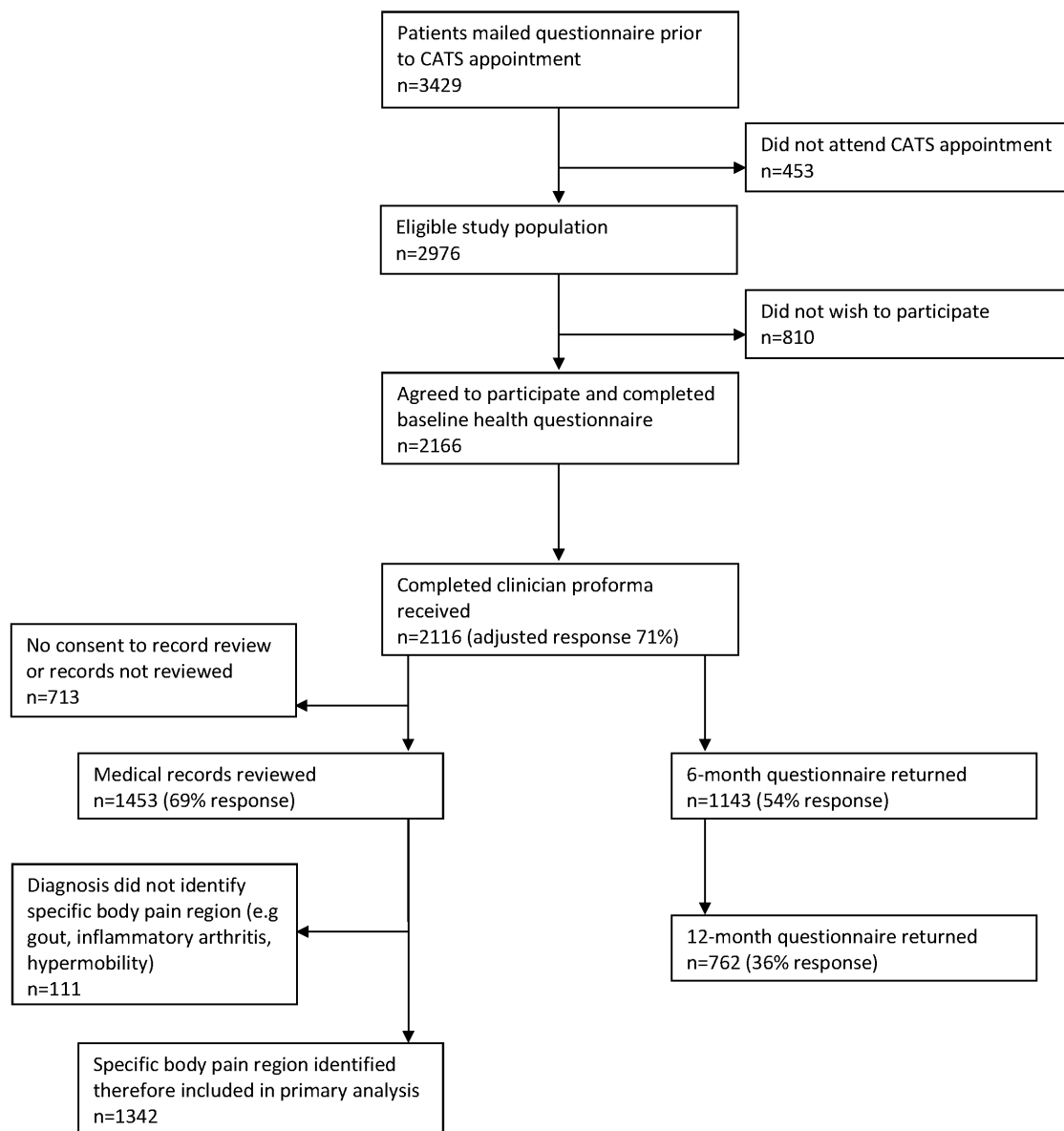

Supplement: supplementary figure — Flow of participants through the study [file bmjopen-2016-011735supp_figure.pdf]
