# Supplementary material for: Reconsultation, self-reported health status and costs following treatment at a musculoskeletal Clinical Assessment and Treatment Service (CATS): a 12-month prospective cohort study
Source: BMJ Open. 2016 Oct 12;6(10):e011735. doi: 10.1136/bmjopen-2016-011735 (PMC5073523; doi:10.1136/bmjopen-2016-011735)
Supplement: supplementary table — comparison of baseline characteristics between those included and excluded from primary care record analysis [file bmjopen-2016-011735supp_table.pdf]

Supplementary table 1 - comparison of baseline characteristics between those included and excluded from primary care record analysis

		Included	Excluded	Difference[a] (95% CI)
n		1453	713	
Female	n (%)	824 (57)	414 (58)	-1.4 (-5.8, 3.1)
Age	Mean (SD)	52.0 (14.9)	49.0 (15.8)	2.6 (1.3, 4.0)[d]
HADS depression	Mean (SD)	6.4 (4.4)	6.2 (4.3)	0.2 (-0.1, 0.6)
HADS anxiety	Mean (SD)	7.9 (4.7)	7.6 (4.6)	0.3 (-0.1, 0.7)
SF36 physical function	Mean (SD)	36.1 (12.1)	37.2 (11.9)	-1.1 (-2.1, 0.03)
SF36 body pain	Mean (SD)	34.1 (8.5)	35.1 (8.8)	-1.1 (-1.8, -0.3)[d]
Pain duration > 1year	n (%)	803 (55)	399 (56)	-0.8 (-5.2, 3.7)
Severe pain interference[b]	n (%)	819 (56)	370 (52)	4.4 (-0.02, 8.9)
Currently employed	n (%)	750 (52)	386 (55)	-2.7 (-7.2, 1.8)
Time off work[c]	n (%)	341 (46)	175 (45)	0.2 (-5.9, 6.3)

CI, confidence interval; HADS, Hospital Anxiety and Depression Scale; SF-36, Short Form-36; SD, standard deviation

[a] difference in means or proportions as appropriate; [b] quite a bit or extremely; [c] due to musculoskeletal problem in the last 6 months in those currently employed; [d] $p<0.05$
